# Immunohistochemical Detection of the Mechano-Gated Piezo Channels in the Normal Endometrium and in Endometriosis

**DOI:** 10.3390/biom16010166

**Published:** 2026-01-19

**Authors:** Angel Sánchez del Rio, Yolanda García-Mesa, Ana Gutiérrez-Palacios, Patricia Cuendias, Eliseo Viña, Graciela Martínez-Barbero, José A. Vega, Olivia García-Suárez

**Affiliations:** 1 Grupo Sistema Nervioso Periférico y Órganos de los Sentidos, Departamento de Morfología y Biología Celular, Universidad de Oviedo, 33006 Oviedo, Spain; angelsanchez@sego.es (A.S.d.R.); garciamyolanda@uniovi.es (Y.G.-M.); cuendiaspatricia@uniovi.es (P.C.); eliseovina@gmail.com (E.V.); gracielamartinezbarbero@gmail.com (G.M.-B.); 2 Instituto de Investigación Sanitaria del Principado de Asturias—ISPA, 33011 Oviedo, Spain; 3 Servicio de Anatomía Patológica, Hospital V. Álvarez-Buylla, 33600 Mieres, Spain; anag.palacios@gmail.com; 4 Servicio de Cardiología, Unidad de Hemodinámica y Cardiología Intervencionista, Hospital Universitario de Cabueñes, 33203 Gijón, Spain; 5 Facultad de Ciencias de la Salud, Universidad Autónoma de Chile, Providencia—Región Metropolitana, Santiago 7500912, Chile

**Keywords:** Piezo1 ion channel, Piezo2 ion channel, healthy endometrium, endometriosis

## Abstract

Endometriosis is an inflammatory estrogen-dependent disorder characterized by pain, dyspareunia, dysmenorrhea, and infertility. This is due to the invasion of different organs by endometrial tissue that causes inflammation, angiogenesis, and fibrosis. The ion channels Piezo1 and Piezo2 primarily work as mechanosensors and mechanotransducers but also have functions that could participate in the clinical hallmarks of endometriosis. Thus, we investigated the occurrence and localization of Piezo1 and Piezo2 in healthy human endometrium and in endometriosis using immunohistochemistry. In healthy endometrium, Piezo1 immunoreactivity was detected in the glands and to a lesser extent in the stroma; Piezo2 was present in the same locations but at low or residual levels. In ectopic endometriosis, there was an increase in the intensity of Piezo1 regardless of location; Piezo2 only showed a net increase in the ovarian and vaginal endometriosis foci. The present results demonstrate the occurrence of Piezo ion channels in the healthy human endometrium for the first time, as well as an increase in Piezo1 in ectopic endometriosis, and no changes in Piezo2 with the exception of the ovary and vagina. However, these results are descriptive and qualitative, although they may serve as the basis for further studies. The role of these ion channels in the endometrium and in the pathogenesis of endometriosis remains to be elucidated, and more precise methods are needed to follow up on this pilot study that can be better analyzed statistically to confirm the results.

## 1. Introduction

Endometriosis is an inflammatory estrogen-dependent disorder that causes chronic pelvic pain, dyspareunia, dysmenorrhea, and infertility, affecting 5–10% of reproductive-age women worldwide [1,2]. The basis of this clinical entity is the invasion of different organs by endometrial-like tissue. This tissue may proliferate and grow in the proper endometrium or outside the uterus (ovary, peritoneum, urinary bladder, or intestine), or more distantly (lung), originating eutopic or ectopic endometriosis, respectively [3,4]. The endometrial tissue infiltration causes inflammation, angiogenesis, fibrosis, the formation of scar tissue, and functional impairments of the host organs [5,6,7]. Nevertheless, despite its high prevalence and impact on quality of life, the pathogenesis of endometriosis is poorly understood [8,9]. Consequently, the available treatment options are limited and mainly focused on symptom management rather than targeting the underlying mechanisms of the disease [10,11,12].

Two emergent ion channels denominated Piezo1 and Piezo2 that primarily work as mechanosensors and mechanotransducers [13,14,15,16] have also been demonstrated to play roles in cellular migration [17], inflammation [18,19,20], angiogenesis [21], fibrosis [22,23,24,25], and pain [26,27,28], which are the primary clinical markers of endometriosis. As far as we know, there is no evidence that Piezo channels have any relation with dysmenorrhea. Thus, Piezo channels are surely more than mechanotransducers or at least they are involved in the linking of forces with different physiological and pathological conditions [29,30,31,32].

Piezo channels are broadly distributed in tissues and cells, predominating Piezo2 in nervous tissues and Piezo1 in non-nervous ones. However, little information is available about the occurrence of Piezo channels in the uterus [33,34]. Therefore, we decided to conduct the present research to detect Piezo1 and Piezo2 in healthy endometrium and to investigate whether their expression changes in eutopic and ectopic endometriosis. We used immunohistochemistry and immunofluorescence coupled with laser confocal microscopy, alongside image analysis.

## 2. Materials and Methods

### 2.1. Subjects

This prospective study enrolled 35 women of which 30 were diagnosed with endometriosis and 5 were controls. The age of patients was 48 ± 9.16 years (mean age ± standard deviation) in the control group, and 35.10 ± 8.3 years in the endometriosis group. All women included in this research were premenopausal in the proliferative phase of the cycle.

Medical records were reviewed to collect relevant clinical information. All patients enrolled in the study were confirmed to be premenopausal with regular menstrual cycles. Endometriosis was surgically and histologically diagnosed as stage I, II, III, or IV according to the revised American Fertility Society (r-AFS) classification. The level of pain was determined using the visual analog scale (VAS), from 0 (no pain) to 10 (worst pain). The VAS scores were grouped into three levels: mild (ranging from 0 to 3), moderate (ranging from 4 to 6), and severe (ranging from 7 to 10). The prevalent symptoms were dysmenorrhea (100%), followed by infertility (55.17%). Intense pain with a visual analog scale value (VAS) > 7 was found in nearly 60% of women. The findings on laparoscopic examination and anatomical pathology demonstrated ovarian endometriosis in 37.93% of cases and peritoneal and intestinal endometriosis in 34.48%, with only a few cases in other localizations (27.59%). Regarding stage, 99.54% of cases corresponded to stage III (33.58%) and IV (68.96%), respectively. All of the signs and symptoms of patients in the endometriosis group are listed in Table 1.

All patients underwent laparoscopy due to symptomatic endometriosis in which the endometriotic tissue was removed. Moreover, endometrium samples from 5 women without endometriosis who had undergone laparoscopy from benign gynecological diseases were used as controls. Subjects with endometrial cyst, pain, infertility, and endometriosis detected through laparoscopic surgery were included in the endometriosis group. Endometriosis was confirmed histologically by the presence of both endometrial glands and stroma in an ectopic location.

### 2.2. Ethics

Tissue samples were obtained at the Service of Pathology of the Hospital Alvarez Buylla, Mieres, Principality of Asturias, Spain. All materials used in the present study were obtained in compliance with Spanish legislation (RD 1301/2006; Law 14/2007; RD 1716/2011; Order ECC/1404/2013) and in accordance with the guidelines of the Declaration of Helsinki II. Informed consent was obtained from patients and the study was approved by the Research Ethics Committee of the Principality of Asturias (Cod CEIm P Ast: Project 266/18).

### 2.3. Treatment of Tissues Samples

The excised tissues from both patients with endometriosis and controls were fixed on 10% formalin (in 1 M PBS, pH 7.4) for 12 h and routinely processes for paraffin embedding. Serial sections 7 µm and 10 µm thick were cut and mounted onto silane-coated slides. Thereafter, sections were deparaffinized and processed for immunohistochemistry or immunofluorescence.

### 2.4. Structural and Immunohistochemical Diagnosis of Endometriosis

To ascertain the diagnosis of endometriosis, representative sections of each specimen were processed as follows: after removing paraffin and permeabilization with 1 M PBS at pH 7.6 with 0.5% Tween-20, the endogenous peroxidase activity was blocked with 10% H_2_O_2_ for 30 min, and non-specific binding was then prevented with 25% fetal bovine serum. Sections were incubated overnight at 4 °C in a humid chamber with the primary antibodies against CD10, the progesterone receptor, and the estrogen receptor (Table 2). Subsequently, the sections were incubated with anti-rabbit or anti-mouse EnVision system-labeled polymer (DakoCytomation, Glostrup, Denmark) for 30 min. Finally, they were washed with buffer solution, and the immunoreaction was visualized with diaminobenzidine as a chromogen. Finally the slides were washed, dehydrated, and mounted with Entellan (Merck, Dramstadt, Germany). To ascertain structural details, the sections were counterstained with Mayer’s hematoxylin. Images of the immunohistochemical results were taken with a Nikon Eclipse^®^ 80i optical microscope (Nikon Europe B.V., Amstelveen, The Netherlands) coupled to a Nokia^®^ DS-5M camera (Nikon Europe B.V., Helsinki, Finland).

CD10 is routinely used in the diagnostic of endometriosis [35,36] and estrogen or progesterone receptors since these hormones promote endometriosis [37,38].

### 2.5. Simple Immunohistochemistry for PIEZOs

Deparaffinized and rehydrated sections were processed as described above and incubated overnight at 4 °C in a humid chamber with the primary antibodies against specific epitopes of Piezo1 and Piezo2 (Table 2). Images of the immunohistochemical results were taken with a Nikon Eclipse^®^ 80i optical microscope coupled to a Nokia^®^ DS-5M camera.

**Table 2:** Primary antibodies used in this study.

Antigen	Host/Origin	Dilution	Supplier
Piezo1 *	Rabbit polyclonal	1:100	Invitrogen ^1^
Piezo2 **	Rabbit polyclonal	1:100	Sigma-Aldrich ^2^
CD10 (SP67)	Rabbit monoclonal	Prediluted	Roche ^3^
anti-Estrogen receptor (ER) (SP1)	Rabbit monoclonal	Prediluted	Roche ^3^
anti-Progesterone receptor (PR) (1E2)	Rabbit monoclonal	Prediluted	Roche ^3^
NFP (2F11)	Mouse monoclonal	1:200	Roche ^3^
CK7	Mouse monoclonal	Prediluted	Dako ^4^
S100P (S100A1)	Rabbit polyclonal	1:500	Dako ^4^

^1^ Waltham, MA, USA; ^2^ St. Louis, MO, USA; ^3^ Barcelona, Spain; ^4^ Glostrup, Denmark. * PIEZO1: Synthetic peptide C-EDLKPQHRRHISIR. ** PIEZO2: Synthetic peptide: VFGFWAFGKHSAAADITSSLSEDQVPGPFLVMVLIKFGTMVLIKFGTMVVDRALYLRK.

### 2.6. Evaluation of Intensity of Immunostaining for PIEZOs and Statistical Analysis

The intensity of immunostaining for Piezo1 and Piezo2 developed in the different tissues was evaluated semi quantitatively as follows: whole section sections were scanned at medium (×50) and high magnification (×200) using an SCN400F scanner (Leica Biosystems™, Newcastle upon Tyne, UK); the images were processed with the SlidePath Gateway LAN program (Leica Biosystems™) at the Histopathology Laboratory, University Institute of Oncology of the Principality of Asturias. The results were evaluated three times by two independent observers (OGS and YGM) blinded to the group of the specimen, i.e., control vs. endometriosis, and the antigen investigated. The results were divided into four groups: − negative, no staining; +: faint; ++: moderate; +++: strong.

On the other hand, the intensity of immunostaining was assessed quantitatively in arbitrary units of gray level ranging from 1 (black) to 256 (white) using the facilities of the Servicios Comunes de Investigación de la Universidad de Oviedo (https://www.sct.uniovi.es/unidades/analisis-biologico/microscopia/equipos, accessed on 10 January 2026). An Olympus BX61 motorized optical microscope (Olympus Corporation, Tokyo, Japan) was used with optics PlanApo 40x/0.95 coupled with an automatic image analysis system (Quantimet 550, Leica, QWIN Program). The system was calibrated taking as “zero” the value of control sections. The levels of sensitivity and light intensity were adjusted to an optimum and kept constant during measurements.

In total, 10 sections of each healthy endometrial or endometriosis sample, 10 μm thick and 50 μm apart, were divided into two sets of 5 sections, processed for immunohistochemical detection of Piezo1 and Piezo2, respectively, and evaluated. In each section, 5 randomly selected fields were evaluated (25 fields per sample and antibody). Data were grouped into four categories (64 units of gray level each) referred to as strong (1–64), high (65–128), intermediate (129–192), and low (193–240) immunoreactivity. Fields showing an intensity of immunostaining > 240 were considered unreactive. The results are expressed as the mean ± SD of the intensity of immunostaining.

For each sample and antibody, immunostaining intensity values obtained from the 25 analyzed microscopic fields were averaged to obtain a single representative value per sample. Microscopic fields and sections were considered technical replicates, and the averaged value per sample was used for all subsequent statistical analyses.

Quantitative immunostaining data from pathological endometrial samples were compared exclusively with those from healthy endometrial tissues. When applicable, each pathological condition was independently compared with the control group. No direct comparisons among different pathological groups were made.

The normality of data distribution was assessed using the Shapiro–Wilk test. For normally distributed data, comparisons were carried out using Student’s *t*-test. When data did not meet normality assumptions, the non-parametric Mann–Whitney *U* test was applied. A *p*-value < 0.05 was considered statistically significant.

### 2.7. Double Immunofluorescence for Identification of Piezo Positive Cells

Deparaffinized and rehydrated sections were washed in PBS-T for 20 min. Then, sections were incubated overnight at 4 °C in a humid chamber with a 1:1 mixture of two antibodies for the simultaneous detection of Piezo1/CK7 and Piezo2/CK7. Subsequently, sections were washed with PBS-T for 30 min and then incubated with the secondary antibodies for 90 min: first, Alexa Fluor 488-conjugated goat anti-rabbit IgG (1:100; Serotec™, Oxford, UK), then washed in PBS-T, and followed by Cy3-conjugated donkey anti-mouse IgG (1:200; Jackson-ImmunoResearch™, Baltimore, MD, USA); both incubations were carried out in a humid chamber, in darkness, and at room temperature. Finally, the sections were stained with DAPI (4′,6-diamino-2-phenylindole; 10 ng/mL) to label the nuclei (blue color) and mounted with diluted Fluoromount-G mounting medium (Southern-Biotech, Birmingham, AL, USA). Triple staining was detected using a Leica DMR-XA automatic fluorescence microscope coupled with Leica Confocal Software, version 2.5 (Leica Microsystems, Heidelberg GmbH, Germany), and the images captured were processed using the software Image J version 1.43 g Master Biophotonics Facility, Mac Master University Ontario (www.macbiophotonics.ca) from the Image Processing Service of the University of Oviedo.

Specific reaction controls were performed in the same way as for simple immunohistochemistry.

For control purposes, selected sections were processed identically as described above omitting the primary and/or secondary antibody in incubation or incubating the sections with non-immune serum from rabbits or mice. Under these conditions, no specific immunoreactivity was found. Additional controls were carried out to confirm the absence of autofluorescence that may interfere with immunofluorescence results.

## 3. Results

### 3.1. Identification of the Endometriosis Foci

**Figure 1:**
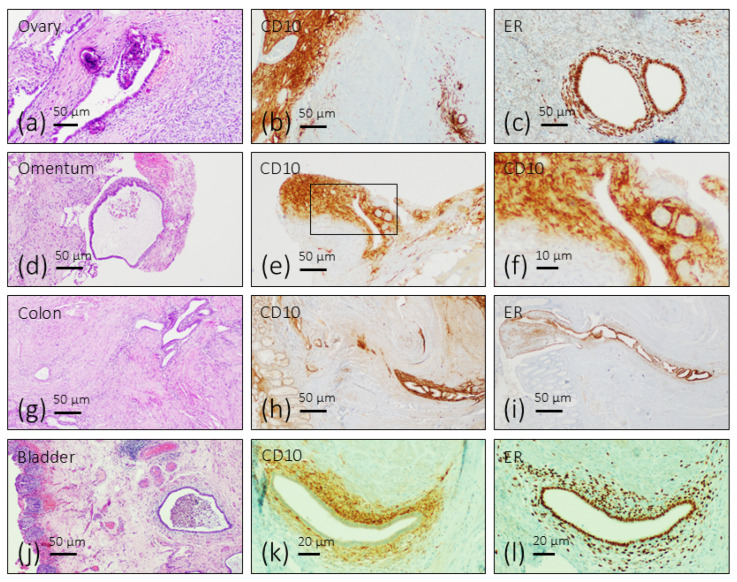
Structural and immunohistochemical identification of endometriosis foci in different organs. Clusters of endometrial glandular epithelium and stroma, occasionally forming cysts, were detected both on the surface and in the depth of the affected organs. The nuclei of the cells of the endometriosis foci were immunoreactive for estrogen and progesterone receptors, and the stroma was immunoreactive for CD10.

Endometriosis foci are characterized by the presence of ectopic endometrial glands surrounded by a fibrous endometrial stroma with inflammatory cells. Occasionally, they form cysts with heterogeneous content including cell debris, traces of hemorrhage, and pigmentation due to hemosiderin deposition (Figure 1a,d,g,j). Regardless of their anatomical location, immunoreactivity for CD10 was detected in the stroma of endometriosis lesions (Figure 1b,e,h,k), and immunoreactivity for estrogen receptors was localized in the nuclei of gland and stromal cells (Figure 1c,f,i,l).

### 3.2. Piezo1 and Piezo2 Immunoreactivity in the Endometrium

**Figure 2:**
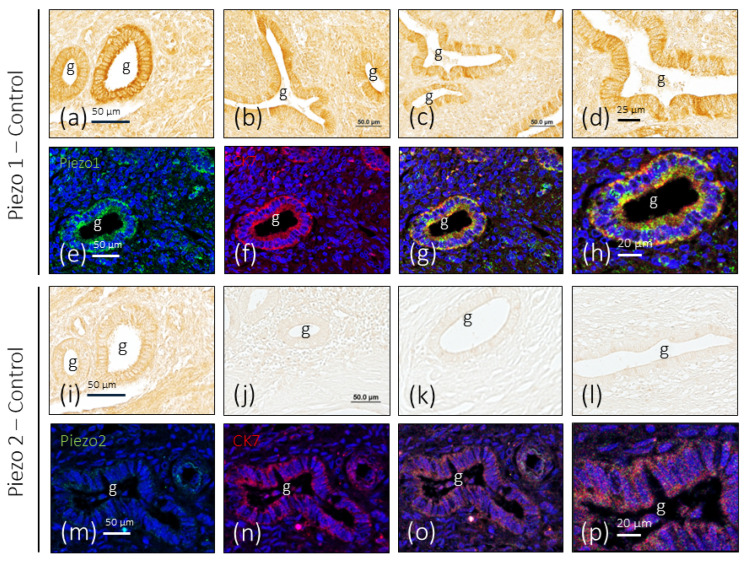
Immunohistochemical localization of Piezo1 and Piezo2 in the healthy endometrium. The healthy endometrium was regarded as the control. Piezo1 immunoreactivity and immunofluorescence were detected in the endometrial glands and stroma (**a**–**h**), while Piezo2 immunoreactivity and immunofluorescence were largely absent or present at residual levels (**i**–**p**). CK7: cytokeratin 7; g: glands. Scale bar in a was identical for (**a**–**d**,**i**–**l**). For images (**e**–**h**,**m**–**p**), objective: 20×/0.75 oil; pinhole: 1; XY resolution: 156 nm; Z resolution: 334 nm.

In endometrial samples obtained from healthy women, immunoreaction for PIEZO1 was regularly detected in the glandular epithelium, with small differences in immunolabeling from one gland to another, and no evident differences were noted between the analyzed samples. The immunolabeling pattern was membrane-shaped, being more intense in the luminal pole of the cells (Figure 2a–d). Doble immunolabeling of the sections to simultaneously detect Piezo1 and cytokeratin 7 (CK7) confirms that Piezo1 immunoreactivity is limited to the cells of the gland epithelium and localized in the cell membrane (Figure 2e–h).

Regarding Piezo2, its presence in the healthy endometrium was restricted to a small number of glands. As for Piezo1, immunoreactivity presented a membrane pattern of immunolabeling (Figure 2i–l) and was restricted to epithelial cells (Figure 2m–p).

When the intensity of immunoreactivity for Piezo and Piezo2 was compared in identically processed serial sections (Figure 1a,i), the difference between the two in favor of Piezo1 can be clearly seen.

### 3.3. Localization of Piezo1 and Piezo2 in Endometriosis

The endometriosis samples analyzed in this study came from endometriosis foci located in the uterus (eutopic endometriosis) or in other organs (ectopic endometriosis). It should be emphasized that the variations in the intensity of immunolabeling observed and reported here in endometriosis are always in relation to the epithelium of healthy endometrial tissue, regarded as the reference control.

#### 3.3.1. Eutopic Endometriosis

**Figure 3:**
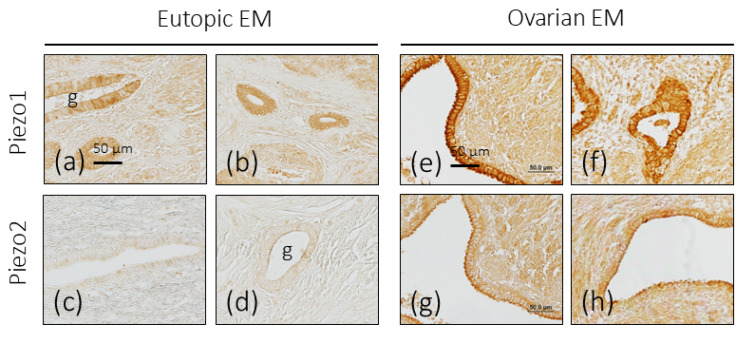
Immunohistochemical localization of Piezo1 and Piezo2 in eutopic and ovarian endometriosis (EM). Piezo1 and Piezo2 immunoreactivity in endometriosis foci in the uterus (**a**–**d**) and ovary (**e**–**h**). g: glands. Scale bar in a is identical for (**a**–**d**) and scale bar in e is identical for (**e**–**h**).

No differences with healthy endometrial tissue were found in eutopic endometriosis for the immunoreactivity of Piezo1 or Piezo2, nor in the number of glands marked, nor in the pattern or intensity of immunolabeling (Figure 3a–d).

#### 3.3.2. Ovarian Endometriosis

In the endometriotic foci placed in the ovary, there was an increase in the intensity of the immunostaining for Piezo1, and only at the level of the luminal pole of the epithelial cells for Piezo2 (Figure 3e–h). In addition, immunoreaction of variable intensity for both mechanoproteins was detected in the stroma surrounding the glands.

#### 3.3.3. Peritoneal Endometriosis

**Figure 4:**
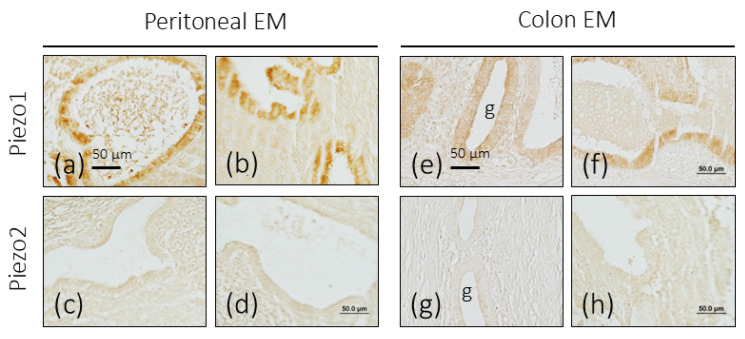
Immunohistochemical localization of Piezo1 and Piezo2 in peritoneal and colon endometriosis (EM). Piezo1 and Piezo2 immunoreactivity in endometriosis foci in the peritoneum (greater omentum) (**a**–**d**) and colon (**e**–**h**). g: glands. Scale bar in a is identical for (**a**–**d**) and scale bar in e is identical for (**e**–**h**).

One of the most common locations of ectopic endometriosis is the peritoneum. In the samples analyzed, there was no evidence of changes in the expression of the immunoreactivity for Piezo2; that is, it was not detected, and that for Piezo1 was higher than that of the control uterine glandular epithelium (Figure 4a–d).

#### 3.3.4. Intestinal Endometriosis

The levels of immunoreaction intensity for Piezo1 and Piezo2 in endometriosis foci in the colon were similar to those observed in the control endometrium (Figure 4e–h).

#### 3.3.5. Vesical Endometriosis

**Figure 5:**
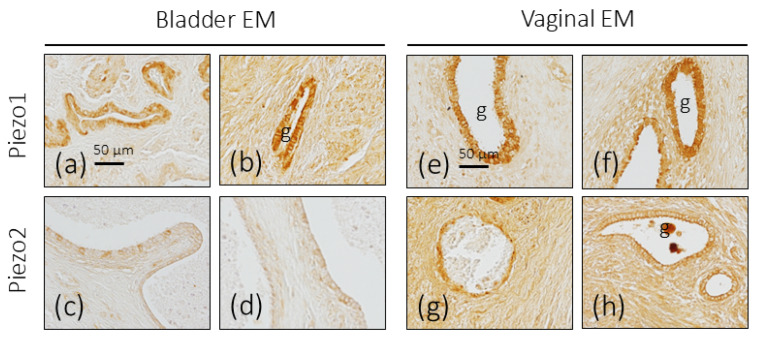
Immunohistochemical localization of Piezo1 and Piezo2 in urinary bladder and vaginal endometriosis (EM). Piezo1 and Piezo2 immunoreactivity in endometriosis foci in the urinary bladder (**a**–**d**) and vagina (**e**–**h**). g: glands. Scale bar in a is identical for (**a**–**d**) and scale bar in e is identical for (**e**–**h**).

In the foci of endometriosis implanted in the urinary bladder, an increase in the intensity of the immunoreaction for Piezo1 was observed both in the glandular cells and in the stroma, especially in the case in which the foci were implanted in the thickness of the detrusor muscle. Regarding Piezo2, it was observed that some isolated epithelial cells had a moderate immunoreaction intensity, with the others being unreactive (Figure 5a–d).

#### 3.3.6. Vaginal Endometriosis

As in the case of the ovary, an increase in the intensity of the immunoreaction for Piezo1 and Piezo2 was observed in both the glandular epithelium and the stroma (Figure 5e–h).

The results of the semi-quantitative study are shown in Table 3 and have been carried out by specialists in the analysis of histological samples processed for the detection of antigens in human tissues fixed and embedded in paraffin. Although the pieces were processed identically, technical factors may have influenced the final result, and it is well known that in immunohistochemistry there is no stoichiometric relationship between antigen quantity and immunoreactivity intensity.

Regarding the results of the quantitative analysis of the intensity of the immunoreaction based on the arbitrary levels of gray in the epithelium, the values obtained fully support direct observations under the microscope and semi-quantitative assemblage. The data are summarized in Table 4. The increases in the intensity of immunoreaction were statistically significant, compared to healthy uterine epithelium, for Piezo1 in the foci of ovarian, bladder, and vaginal endometriosis, and for Piezo2 in the foci of ovarian and vaginal endometriosis.

## 4. Discussion

Piezo1 and Piezo2 ion channels were originally described as mediators of mechanotransduction [13], but it is now accepted that they participate in multiple physiological and pathophysiological processes involving forces such as touch, proprioception, pain, angiogenesis, tissue remodeling, fibrosis, and inflammation [14,16,22,39,40,41]. On the other hand, it is well known that dysregulation of Piezo1 function is associated with fibrotic disorders [42], chronic pain syndromes and inflammatory diseases [18], and cancer [43]. Furthermore, mutations in the genes encoding Piezo1 and Piezo2 are responsible for multiple hereditary human diseases [44]. Therefore, the Piezo channels are linked to a wide range of diseases but as far as we know there is no data linking them to endometriosis.

In the present research, the expressions of Piezo1 and Piezo2 in normal endometrium and in eutopic and ectopic endometriosis have been studied using immunohistochemistry. Our results show that in normal healthy endometrium, Piezo1 and Piezo2 are expressed in the epithelial cells of the glands—although the immunoreactivity for Piezo1 is much more potent than for Piezo2. Numerous studies using different techniques have shown the expression of both ion channels in epithelial cells, for example, in the breast, skin, intestine, etc. [13,45,46,47,48]. Our results extend the expression of these channels to the epithelium of the human uterus, most notably Piezo1. We also observe immunoreactivity for Piezo1 and Piezo2 in the smooth muscle cells of the blood vessels and in the myometrium, but always at lower levels than in the epithelial tissue. Previous studies have already demonstrated the presence of Piezo1 mRNA in primary human endometrial epithelial and stromal cells. Moreover, consistent with previous research which observed low levels of Piezo2 mRNA in human and murine endometrial epithelial and stromal cells [33], we observed weak or negative staining for Piezo2 in some endometrial glands. Additionally, we detected Piezo1 staining in human endometrial blood vessels, aligning with earlier findings of Piezo1 expression in rat uterine blood vessels [34]. Mechanical forces influence endometrial cell behavior throughout the menstrual cycle, preparing the endometrium for embryo implantation [49] and acting through various types of mechanosensitive channels [50].

In general, endometriosis produced slight-to-moderate increases in Piezo1 and no changes in Piezo2 expression. However, in the endometriosis foci found in the ovary and vagina, the immunoreactivity for both ion channels increased markedly. Thus, the organs where the increase in the expression of Piezo1 and Piezo2 was most evident were those with well-known hormonal regulation. As far as we know, the possible hormonal regulation of the expression of Piezo channels in the uterus has not been demonstrated, and even less so in endometriosis. However, it is known in other organs under hormonal control such as the breast that Piezo2 expression positively correlates with the estrogen receptor (ER) and progesterone receptor [51], although the signaling pathways are still unknown.

Endometriosis is a chronic inflammatory condition characterized by the presence of endometrial-like tissue within or outside the uterus, resulting in pelvic pain and infertility [52]. Piezo channels could participate in all the hallmarks of endometriosis, i.e., pain, fibrosis, and inflammation [16]. For example, in endometriosis there is frequently fibrotic tissue inside and around the glandular foci, especially in stages III and IV, which are the majority of our cases, and Piezo1 is related to fibrosis [23]. In addition, endometriosis causes inflammation and both Piezo1 and Piezo2 promote pro-inflammatory signaling [53,54] and inflammation-related pain states [55,56].

Understanding the roles of Piezo1 and Piezo2 in endometriosis could provide novel insights into the disease’s pathogenesis and identify new molecular targets for therapeutic intervention. Recent advances in Piezo channel pharmacology [57,58,59] make this an especially timely and relevant avenue of research. Such studies could pave the way for more effective treatments that address the mechanobiological underpinnings of this debilitating condition, improving patient outcomes.

Various limitations to this study should be considered, especially the size of the sample, the organs where the foci of endometriosis are located, and that only one study technique is used. Furthermore, the data presented are mainly qualitative. We consider this work to be the basis for further studies using more precise methods, especially molecular biology techniques (pRT-PCR and Western blot) and functional studies using agonists such as YODA or antagonists (GSMtx4) and a larger number of samples and locations to confirm the present data. However, the data still demonstrate a tendency for increased Piezo1 in endometriosis and point to hormonal regulation of the expression of Piezo1 and Piezo2, although these data must be better analyzed statistically to confirm the results. Taken together, this work provides a descriptive framework for the presence and distribution of Piezo1 and Piezo2 channels in the human endometrium and endometriosis and establishes a morphological basis for future investigations. Further studies incorporating quantitative molecular analysis and functional assays will be necessary to determine whether Piezo channels play an active role in the pathophysiology of endometriosis. Despite these limitations, our findings provide valuable evidence of the relationship between Piezo channels and the hallmarks of endometriosis.

## 5. Conclusions

As a whole, the present results demonstrate for the first time the occurrence of Piezo1, and with less expression Piezo2, in healthy human endometrium, as well as an increase in Piezo1 in ectopic endometriosis, regardless of the anatomical location of the endometriosis foci. Piezo2 is only evidently increased in the foci of endometriosis of the ovary and vagina, which suggests hormonal control of its expression—although future studies are necessary to prove or reject this.

## Figures and Tables

**Table 1:** Relevant clinical data from patients with endometriosis.

**Age** (mean ± SD)	35.10 ± 8.30	
**Chief complaint**	Dysmenorrhea	30 (100%)
	Dyspareunia	7 (23.3%)
	Dyschezia	6 (20%)
	Dysuria	4 (13.3%)
	Sterility	16 (53.3%)
**Visual analog scale (VAS)**	0–3	3 (10%)
	4–7	10 (33.3%)
	>7	17 (56.66%)
**Laparoscopic and pathological findings**	Ovarian endometriosis	10 (37.93%)
	Peritoneal and digestive endometriosis	10 (34.48%)
	Other endometriosis localization (vesical, vaginal, abdominal wall)	5 (13.79%)
	Eutopic endometrium from endometriosis	5 (13.79%)
**Staging of endometriosis**	III	8 (26.66%)
	IV	22 (73.33%)

**Table 3:** Results of the semi-quantitative study on the intensity of immunoreactivity for Piezo1 and Piezo2 in the glandular epithelium and stroma of the samples of healthy uterus and endometriosis studied. − negative, no staining; +: faint; ++: moderate; +++: strong. EM endometriosis. The cubes marked in gray are those that offer clearly different results compared to the controls.

Antigen	Piezo 1	Piezo 2	Stroma P1/P2
Endometrium	+/++	−/+	+/+
Eutopic EM	+/++	−/+	+/−
Ovarian EM	++/+++	++	++/++
Peritoneal EM	+/++	−/+	+/−
Intestinal EM	++	−/+	
Vesical EM	+++	−/+	
Vaginal EM	++/+++	++	++/++

**Table 4:** Results of the quantitative study on the intensity of immunoreactivity for Piezo1 and Piezo2 in the glandular epithelium of the samples of healthy uterus and endometriosis studied. Results are expressed as values of mean ± standard deviation of arbitrary units of gray level: strong (1–64), high (65–128), intermediate (129–192), and low (193–240) immunoreactivity. The cubes marked in red are those offering significant differences with respect compared to the controls. * *p*-value < 0.05.

Antigen	Piezo 1	Piezo 2
Endometrium	178.3 ± 5.25	201.2 ± 6.6
Eutopic EM	161.3 ± 10.2	198.1 ± 8.2
Ovarian EM	79.2 ± 8.1 *	132.6 ± 6.8 *
Peritoneal EM	193.1 ± 14.2	213.7 ± 14.3
Intestinal EM	129.1 ± 16.2	211.4 ± 16.1
Vesical EM	47.3 ± 3.3 *	223.8 ± 7.9
Vaginal EM	85.1 ± 9.4 *	126.2 ± 16.9 *

## Data Availability

The data that support the findings of this study are available from the corresponding authors upon reasonable request. The data are anonymized and there are no ethical or contractual restrictions on its use. They are also not subject to intellectual property.

## References

[1] As-Sanie S., Black R., Giudice L.C., Gray Valbrun T., Gupta J., Jones B., Laufer M.R., Milspaw A.T., Missmer S.A., Norman A. (2019). Assessing research gaps and unmet needs in endometriosis. Am. J. Obstet. Gynecol..

[2] As-Sanie S., Mackenzie S.C., Morrison L., Schrepf A., Zondervan K.T., Horne A.W., Missmer S.A. (2025). Endometriosis: A Review. JAMA.

[3] Vannuccini S., Clemenza S., Rossi M., Petraglia F. (2022). Hormonal treatments for endometriosis: The endocrine background. Rev. Endocr. Metab. Disord..

[4] Bo C., Wang Y. (2024). Angiogenesis signaling in endometriosis: Molecules, diagnosis and treatment (Review). Mol. Med. Rep..

[5] Gruber T.M., Mechsner S. (2021). Pathogenesis of Endometriosis: The Origin of Pain and Subfertility. Cells.

[6] Velho R.V., Taube E., Sehouli J., Mechsner S. (2021). Neurogenic Inflammation in the Context of Endometriosis-What Do We Know?. Int. J. Mol. Sci..

[7] Mariadas H., Chen J.H., Chen K.H. (2025). The Molecular and Cellular Mechanisms of Endometriosis: From Basic Pathophysiology to Clinical Implications. Int. J. Mol. Sci..

[8] Wang Y., Nicholes K., Shih I.M. (2020). The Origin and Pathogenesis of Endometriosis. Annu. Rev. Pathol. Mech. Dis..

[9] Kobayashi H., Imanaka S., Yoshimoto C., Matsubara S., Shigetomi H. (2024). Rethinking the pathogenesis of endometriosis: Complex interactions of genomic, epigenetic, and environmental factors. J. Obstet. Gynaecol. Res..

[10] Parasar P., Ozcan P., Terry K.L. (2017). Endometriosis: Epidemiology, Diagnosis and Clinical Management. Curr. Obstet. Gynecol. Rep..

[11] Kou L., Huang C., Xiao D., Liao S., Li Y., Wang Q. (2025). Pharmacologic Interventions for Endometriosis-Related Pain: A Systematic Review and Meta-analysis. Obstet. Gynecol..

[12] Mick I., Freger S.M., van Keizerswaard J., Gholiof M., Leonardi M. (2024). Comprehensive endometriosis care: A modern multimodal approach for the treatment of pelvic pain and endometriosis. Ther. Adv. Reprod. Health.

[13] Coste B., Mathur J., Schmidt M., Earley T.J., Ranade S., Petrus M.J., Dubin A.E., Patapoutian A. (2010). Piezo1 and Piezo2 are essential components of distinct mechanically activated cation channels. Science.

[14] Murthy S.E., Dubin A.E., Patapoutian A. (2017). Piezos thrive under pressure: Mechanically activated ion channels in health and disease. Nat. Rev. Mol. Cell Biol..

[15] Delmas P., Parpaite T., Coste B. (2022). PIEZO channels and newcomers in the mammalian mechanosensitive ion channel family. Neuron.

[16] Xiao B. (2024). Mechanisms of mechanotransduction and physiological roles of PIEZO channels. Nat. Rev. Mol. Cell Biol..

[17] Sforna L., Michelucci A., Morena F., Argentati C., Franciolini F., Vassalli M., Martino S., Catacuzzeno L. (2022). Piezo1 controls cell volume and migration by modulating swelling-activated chloride current through Ca^2+^ influx. J. Cell. Physiol..

[18] Tang Y., Zhao C., Zhuang Y., Zhong A., Wang M., Zhang W., Zhu L. (2023). Mechanosensitive Piezo1 protein as a novel regulator in macrophages and macrophage-mediated inflammatory diseases. Front. Immunol..

[19] Du S., Liu K. (2025). Mechanosensitive ion channels and inflammation: Key links in cellular signal transduction. Inflamm. Res..

[20] Pirri C. (2025). PIEZO Channels in Mechano-Inflammation: Gatekeepers of Neuroimmune Crosstalk. Diseases.

[21] Alibrandi S., Rinaldi C., Vinci S.L., Conti A., Donato L., Scimone C., Sidoti A., D’Angelo R. (2025). Mechanotransduction in Development: A Focus on Angiogenesis. Biology.

[22] Di X., Gao X., Peng L., Ai J., Jin X., Qi S., Li H., Wang K., Luo D. (2023). Cellular mechanotransduction in health and diseases: From molecular mechanism to therapeutic targets. Signal Transduct. Target. Ther..

[23] Xu Y., Huang Y., Cheng X., Hu B., Jiang D., Wu L., Peng S., Hu J. (2023). Mechanotransductive receptor Piezo1 as a promising target in the treatment of fibrosis diseases. Front. Mol. Biosci..

[24] Zheng M., Borkar N.A., Yao Y., Ye X., Vogel E.R., Pabelick C.M., Prakash Y.S. (2023). Mechanosensitive channels in lung disease. Front. Physiol..

[25] Drobnik M., Smólski J., Grądalski Ł., Niemirka S., Młynarska E., Rysz J., Franczyk B. (2024). Mechanosensitive Cation Channel Piezo1 Is Involved in Renal Fibrosis Induction. Int. J. Mol. Sci..

[26] Wan Y., Zhou J., Li H. (2024). The Role of Mechanosensitive Piezo Channels in Chronic Pain. J. Pain Res..

[27] Xu Y., Wang Y., Mei S., Hu J., Wu L., Xu L., Bao L., Fang X. (2024). The mechanism and potential therapeutic target of piezo channels in pain. Front. Pain Res..

[28] Liu C., Li H., Hang L. (2025). The research progress into cellular mechanosensitive ion channels mediating cancer pain. Channels.

[29] Burridge K., Monaghan-Benson E., Graham D.M. (2019). Mechanotransduction: From the cell surface to the nucleus via RhoA. Philos. Trans. R. Soc. Lond. B Biol. Sci..

[30] Martino S. (2023). Mechanobiology in Cells and Tissues. Int. J. Mol. Sci..

[31] Martino F., Perestrelo A.R., Vinarský V., Pagliari S., Forte G. (2018). Cellular Mechanotransduction: From Tension to Function. Front. Physiol..

[32] Lacroix J.J., Wijerathne T.D. (2025). PIEZO channels as multimodal mechanotransducers. Biochem. Soc. Trans..

[33] Hennes A., Held K., Boretto M., De Clercq K., Van den Eynde C., Vanhie A., Van Ranst N., Benoit M., Luyten C., Peeraer K. (2019). Functional expression of the mechanosensitive PIEZO1 channel in primary endometrial epithelial cells and endometrial organoids. Sci. Rep..

[34] Arishe O.O., Ebeigbe A.B., Webb R.C. (2020). Mechanotransduction and Uterine Blood Flow in Preeclampsia: The Role of Mechanosensing Piezo 1 Ion Channels. Am. J. Hypertens..

[35] Toki T., Shimizu M., Takagi Y., Ashida T., Konishi I. (2002). CD10 is a marker for normal and neoplastic endometrial stromal cells. Int. J. Gynecol. Pathol..

[36] Potlog-Nahari C., Feldman A.L., Stratton P., Koziol D.E., Segars J., Merino M.J., Nieman L.K. (2004). CD10 immunohistochemical staining enhances the histological detection of endometriosis. Fertil. Steril..

[37] Chantalat E., Valera M.C., Vaysse C., Noirrit E., Rusidze M., Weyl A., Vergriete K., Buscail E., Lluel P., Fontaine C. (2020). Estrogen Receptors and Endometriosis. Int. J. Mol. Sci..

[38] Coroleucă C.A., Coroleucă C.B., Coroleucă R., Brătilă P.C., Nodiți A.R., Roșca I., Brîndușe L.A., Brătilă E., Boț M. (2025). Molecular Profile (Estrogen Receptor, Progesterone Receptor, Bcl-2 and Ki-67) of the Ectopic Endometrium in Patients with Endometriosis. Int. J. Mol. Sci..

[39] Syeda R. (2021). Physiology and Pathophysiology of Mechanically Activated PIEZO Channels. Annu. Rev. Neurosci..

[40] Poole K. (2022). The Diverse Physiological Functions of Mechanically Activated Ion Channels in Mammals. Annu. Rev. Physiol..

[41] Xu X., Liu S., Liu H., Ru K., Jia Y., Wu Z., Liang S., Khan Z., Chen Z., Qian A. (2021). Piezo Channels: Awesome Mechanosensitive Structures in Cellular Mechanotransduction and Their Role in Bone. Int. J. Mol. Sci..

[42] Liu X., Niu W., Zhao S., Zhang W., Zhao Y., Li J. (2023). Piezo 1: The potential new therapeutic target for fibrotic disease. Prog. Biophys. Mol. Biol..

[43] Karska J., Kowalski S., Saczko J., Moisescu M.G., Kulbacka J. (2023). Mechanosensitive Ion Channels and Their Role in Cancer Cells. Membranes.

[44] Alper S.L. (2017). Genetic Diseases of PIEZO1 and PIEZO2 Dysfunction. Curr. Top. Membr..

[45] Piddini E. (2017). Epithelial Homeostasis: A Piezo of the Puzzle. Curr. Biol..

[46] García-Mesa Y., Cuendias P., Alonso-Guervós M., García-Piqueras J., Martín-Biedma B., Cobo T., García-Suárez O., Vega J.A. (2024). Immunohistochemical detection of PIEZO1 and PIEZO2 in human digital Meissner’s corpuscles. Ann. Anat. Anat. Anz..

[47] He H., Zhou J., Xu X., Zhou P., Zhong H., Liu M. (2024). Piezo channels in the intestinal tract. Front. Physiol..

[48] Pyo I.H., Yoon Y.B., Jeong G.H., Park S.C., Lee G.W., Aryal Y.P., Kwak H.J., Cho S.J. (2025). Unveiling salivary gland-specific gene expression of Piezo1 and Neuroendocrine in the leech, Helobdella austinensis. Dev. Comp. Immunol..

[49] Sternberg A.K., Buck V.U., Classen-Linke I., Leube R.E. (2021). How Mechanical Forces Change the Human Endometrium during the Menstrual Cycle in Preparation for Embryo Implantation. Cells.

[50] Davoodi Nik B., Hashemi Karoii D., Favaedi R., Ramazanali F., Jahangiri M., Movaghar B., Shahhoseini M. (2024). Differential expression of ion channel coding genes in the endometrium of women experiencing recurrent implantation failures. Sci. Rep..

[51] Lou W., Liu J., Ding B., Jin L., Xu L., Li X., Chen J., Fan W. (2019). Five miRNAs-mediated PIEZO2 downregulation, accompanied with activation of Hedgehog signaling pathway, predicts poor prognosis of breast cancer. Aging.

[52] Zondervan K.T., Becker C.M., Missmer S.A. (2020). Endometriosis. N. Engl. J. Med..

[53] Xie Y., Hang L. (2024). Mechanical gated ion channel Piezo1: Function, and role in macrophage inflammatory response. Innate Immun..

[54] Dubin A.E., Schmidt M., Mathur J., Petrus M.J., Xiao B., Coste B., Patapoutian A. (2012). Inflammatory signals enhance piezo2-mediated mechanosensitive currents. Cell Rep..

[55] Szczot M., Liljencrantz J., Ghitani N., Barik A., Lam R., Thompson J.H., Bharucha-Goebel D., Saade D., Necaise A., Donkervoort S. (2018). PIEZO2 mediates injury-induced tactile pain in mice and humans. Sci. Transl. Med..

[56] Romero L.O., Caires R., Nickolls A.R., Chesler A.T., Cordero-Morales J.F., Vásquez V. (2020). A dietary fatty acid counteracts neuronal mechanical sensitization. Nat. Commun..

[57] De Logu F., Geppetti P. (2019). Ion Channel Pharmacology for Pain Modulation. Concepts and Principles of Pharmacology. Handbook of Experimental Pharmacology.

[58] Kinsella J.A., Debant M., Parsonage G., Morley L.C., Bajarwan M., Revill C., Foster R., Beech D.J. (2024). Pharmacology of PIEZO1 channels. Br. J. Pharmacol..

[59] Thien N.D., Hai-Nam N., Anh D.T., Baecker D. (2024). Piezo1 and its inhibitors: Overview and perspectives. Eur. J. Med. Chem..

